# Valorization of Second-Grade Date Fruit Byproducts and Nonstandard Sweet Potato Tubers to Produce Novel Biofortified Functional Jam

**DOI:** 10.3390/foods12091906

**Published:** 2023-05-06

**Authors:** Bayan J. Almaghlouth, Nashi K. Alqahtani, Khadijah I. Alnabbat, Hisham A. Mohamed, Tareq M. Alnemr, Hosam M. Habib

**Affiliations:** 1 Department of Food and Nutrition Sciences, College of Agricultural and food Sciences, King Faisal University, Al Hufuf 31982, Saudi Arabia; 220002866@student.kfu.edu.sa (B.J.A.); nalqahtani@kfu.edu.sa (N.K.A.); talnemr@kfu.edu.sa (T.M.A.); 2 Date Palm Research Center of Excellence, King Faisal University, P.O. Box 400, Al-Ahsa 31982, Saudi Arabia; hamohammed@kfu.edu.sa; 3 Central Laboratory for Date Palm Research and Development, Agriculture Research Center, Giza 12511, Egypt; 4 Research & Innovation Hub, Alamein International University (AIU), Alamein City 5060310, Egypt

**Keywords:** date fruit, sweet potato, mixed fruit jam, valorization byproducts, functional food

## Abstract

Byproducts of second-grade dates and sweet potato tubers of noncommercial standard are produced along with the main product and are just as important as the main product but cannot be sold in the open market, as they may not be considered acceptable by consumers. Such byproducts can be valorized through the manufacture of a wide range of functional food products with high market appeal, such as jams. The research approach of this study included measuring antioxidant activity, total flavonoids, polyphenols, physicochemical and color indices, pH, and total sugar, as well as conducting a sensory evaluation, of mixed jams composed of different ratios of date jam (DFJ) to sweet potato jam (SPJ), namely, DP1 (80:20), DP2 (70:30), DP3 (60:40), and DP4 (50:50). To date, no other studies have considered producing mixed jam from dates and sweet potato byproducts. The sensory evaluation results indicated that jam DP4 (consisting of 50% date and 50% sweet potato) had the maximum overall acceptability. This investigation reveals the potential of using mixed byproducts in jams as natural functional ingredients, suggesting the economic value of valorization byproducts as low-cost ingredients to expand the properties, nutritional value, antioxidant content, and overall acceptability of jams. The discovered optimal mixed fruit jam has significant potential for further development as a commercial product.

## 1. Introduction

The valorization of post-harvest and food manufacturing byproducts has captured the widespread attention of researchers, due to their potential to promote the sustainability of innovative and novel functional foods [1,2,3,4,5]. Biofortified fruits increase the number of important micronutrients in fortified foods, enhancing their nutritional value and providing extra health profits at no added risk [6,7]. Biofortification acts as a vehicle to further incorporate nutrients, accompaniments, and/or preservatives that are present in low quantities or absent in the food matrix [5,8]. Biofortification prevents nutrient insufficiencies and accompanying issues, helping to stabilize the nutrient profile and reinstate nutrients lost through processing, satisfying and gaining the acceptance of consumers looking to enhance their diets [5]. Biofortification of fruits has also been considered as a public health approach, in order to increase nutrient consumption in the population [9].

The Statistics Division of the Food and Agriculture Organization of the United Nations [10] described the worldwide production of date fruit (*Phoenix dactylifera* L.) as about 9.5 million tons every year [11]. Date fruit harvest leads to the production of huge amounts of date byproducts. In fact, around 30% of dates are generally wasted or used as animal feedstuff, due to undesirable texture (i.e., too hard or too soft), insect infestation, fungal contamination, or low value and quality [12]. The use of such byproducts for limited determinations comprises a significant economic loss, as they have great potential as a promising raw material for the abstraction of bioactive compounds and the creation of value-added resources [13]. The valorization of rejected second-grade date fruit for the manufacture of low-cost value-added products can benefit environmental balance-upsetting problems and inspire industrial bodies to encourage a global perception concerning the utilization and production of these ingredients. In this respect, researchers have broadly studied the characterization and extraction of several segments with high added value—including pectin, fibers, proteins, oils, sugars, and gums—from second-grade dates [14].

Sweet potato is one of the seven main essential crops globally and is presently cultivated in more than 100 countries. It plays a significant role in developing countries, due to its hardiness, low input necessities, and adaptability [15]. Sweet potatoes (*Ipomoea batatas* L.) are a source of phytochemicals, β carotenoids, vitamin A, and minerals which are considered significant due to their valuable effects when included in the diet [16]. One of the difficulties faced by growers around the world is post-harvest byproducts. Regarding sweet potato, parts of the tubers are regularly discarded due to their bad development, appearance, and size. Nevertheless, they remain in perfect condition for consumption by humans and are regularly free from injuries. Furthermore, the use of this type of sweet potato tuber will contribute to reducing the byproduct volume, representing a socially and economically sustainable choice [17].

The processing of fruits or byproducts into jam can greatly reduce post-harvest losses [18]. Jams are among the most prevalent and widespread preserved fruit products, which are made by heating the fruit’s flesh with sugar, citric acid, pectin, and/or other components, such as dyes, vitamin C, preservatives, and flavorings, until a rich and semisolid uniformity is attained [19]. Consequently, this research aims to investigate the possibility of using second-grade date and nonstandard sweet potato fruit as raw materials for producing novel functional jams with high nutritional and antioxidant value. Furthermore, we evaluate the physicochemical and sensory properties of mixed fruit jams in comparison with the individual fruit jams. In this way, we intend to provide a solution for valorizing the byproducts of date fruit and sweet potato, which are usually discarded or underutilized.

## 2. Materials and Methods

### 2.1. Materials

#### 2.1.1. Plant Material

Second-grade date fruits (*Phoenix dactylifera* L., cv. ‘Khalas’) were used at the full ripening stage (Tamar) at the end of the collection season and were obtained from the local date processing industry of Al-Ahasa Eastern Province, Saudi Arabia. Ripe sweet potato (*Ipomoea batatas* L., cv. ‘Beauregard’) nonstandard tubers with orange flesh were selected and harvested freshly from an organic farm at the Station of Research and Training, King Faisal University, Al-Hasa, Saudi Arabia.

#### 2.1.2. Study Materials

The sugar (white sugar; sucrose) was obtained from local supplier markets, Al-Hasah, Eastern Province, K.S.A. Citric acid and pectin powder additives were obtained from iHerb for Electronic Trading and Supplies, headquarters: Morenovalle, CA, USA, which certified that the additives were made from natural, non-GMO, gluten-free food ingredients. Acetonitrile, ethyl acetate, methanol (HPLC grade), 2,4,6-tri(2-pyridyl)-s-triazine (TPTZ), naphthyl ethylene, 1,1-diphenyl-2-picrylhydrazyl (DPPH^●^), FeSO_4_, HCL, AlCl_3_, Folin–Ciocâlteu’s phenol reagent, NaNO_2_, NaOH, and β-carotene were purchased from Sigma-Aldrich Chemical Co. (St. Louis, MO, USA). Standard solution (1 g/L) (calcium (Ca), sodium (Na), potassium (K), phosphorus (P), magnesium (Mg), iron (Fe), and zinc (Zn)) was purchased from Merck (Darmstadt, Germany). Transparent glass cans with screw caps, with a capacity of 150 g of paste, were bought from local supplier markets, Al-Hasah, Eastern Province, K.S.A.

### 2.2. Methods

#### 2.2.1. Preparation of Paste of Date Fruits and Sweet Potato Tubers

Date fruits and sweet potato paste were prepared by optimizing the amount of water used in the cooking process to reduce the flesh/water ratio, reduce the cooking time, and increase the total soluble solids (Brix %).

#### 2.2.2. Preparation of Date Fruit Paste

Date fruits of the Khalas variety were washed under tap water to remove dirt and dust from the fruit’s skin and were then deseeded. Next, the flesh was chopped into 0.5–1.0 cm pieces using a shredder. To prepare the date paste by cooking at 60–65 °C, water was added to the flesh at a ratio of 1:1 *w*/*w*. The mixture was gently blended until a softened texture was observed, and the total soluble solids (TSSs, Brix, using a refractometer) and cooking time were registered.

#### 2.2.3. Preparation of Sweet Potato Tuber Paste

After cleaning and washing, the sweet potato tubers were peeled and weighed. To prepare the tuber flesh by cooking at 60–65 °C, water was added to the flesh at a ratio of 1:1 *w*/*w*, as described previously [20]. The mixing process continued for 15 min to obtain a softened texture, after which the flesh of the tuber was blended using an electric blender. Lastly, a refractometer was used to measure the blend’s total soluble solids (TSSs, Brix).

#### 2.2.4. Combining the Sweet Potato Tuber and Date Fruit Pastes

**Figure 1:**
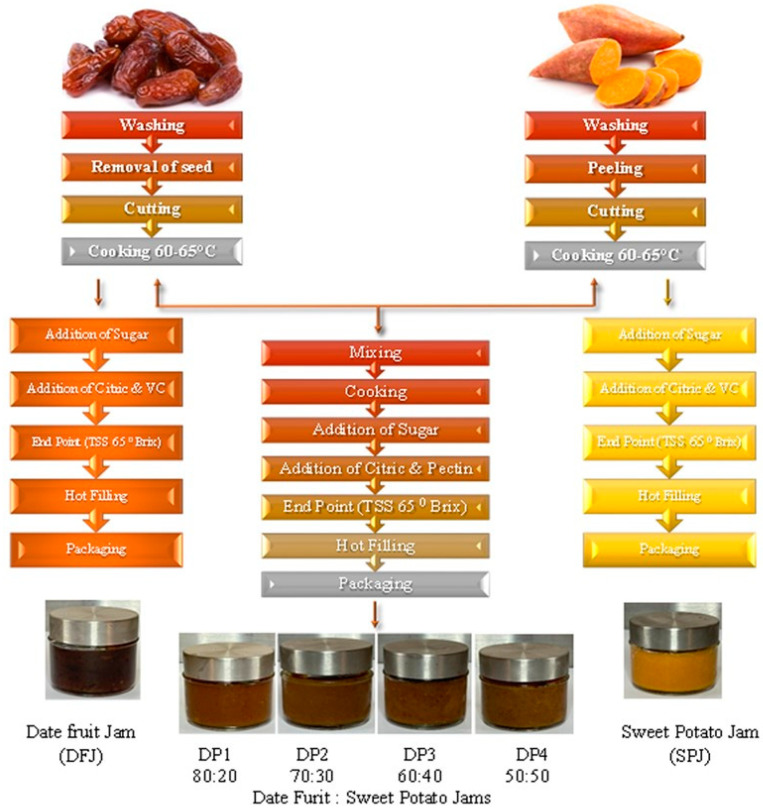
Flowchart of manufacturing process using date fruit and sweet potato pastes.

To study the effects of the sweet potato paste combined with date fruit paste on the physiochemical and sensory properties of the jam, various ratios of the two pastes were tested to determine the optimal combination. Date fruit paste was combined with sweet potato paste in different ratios (80:20, 70:30, 60:40, and 50:50). The combined pastes were blended well using an electric mixer, and we calculated the required amount of white sugar (sucrose) to be added to obtain a final jam product concentration up to 65% ± 2% in the combined ratio, as detailed in Table 1 and Figure 1.

### 2.3. Physiochemical Properties of Date Fruit and Sweet Potato Tubers

#### 2.3.1. pH

A pH meter (Benchtop pH Meter, H1110) was used for pH analysis, in order to measure the pH values of fruits and of each prepared jam treatment after calibrating with a standard buffer solution of pH 7 or 4, at or below 25 °C. The readings were taken immediately after opening the can.

#### 2.3.2. Total Soluble Solids (TSSs)

The total soluble solids (TSSs) were calculated from the absorbance values measured using an Abbey device (Milton Roy, USA; located at 1701 West Main Street, Radnor, PA 19087) according to the following equation: TSS = (absorbance at 540 nm–absorbance at 700 nm) × 0.5. This device is capable of measuring absorbance values in the range of 0.0001 to 5.0 AU, using a xenon lamp as a light source with a spectral range of 190–1100 nm, as described previously [20].

#### 2.3.3. Color Measurement

Observed color measurements were established using a Hunter lab colorimeter (Model D 25-2 Hunter lab, Hunter Reston, VA, USA), where lightness (L*), redness (a*), yellowness (b*), hue angle (°h), and chroma (C*) are the established color parameters using the D65 illuminant and the 10° standard observer. The lightness parameter varies from 0 (black) to 100 (white), while the redness and yellowness parameters vary from negative values (greenness and blueness, respectively) to positive values (redness and yellowness, respectively). The C* value indicates the chroma or saturation of the color, which is calculated as the square root of a^2^ + b^2^. The °h value indicates the hue. The CIE 1976 (Lab*) color system, an internationally accepted system of color measurement, was used as the basis for the color parameters [21].

#### 2.3.4. Moisture Content

All tested samples were analyzed for moisture content in a moisture balance with a halogen lamp heating division (MS-70, A&D Instruments Company Ltd., Abingdon, UK), exposed to step heating up to 130 °C. Persistent weight was expanded at ±0.02% precision [5].

#### 2.3.5. Total Sugars

Total sugars were determined according to the methods of the AOAC (2012) [22].

#### 2.3.6. Total Protein

Total protein was measured by following a previously described method [23], using a Kjeldahl semiautomated Foss model 2300 (Foss Tecator, Hoganas, Sweden).

#### 2.3.7. Ash

Ash contents were measured by carbon exclusion of 2 g of sample, which was burned and incinerated in a muffle furnace at 555 °C for 2 h. The bottle was then removed from the heat and left to cool down. Four milliliters of H_2_O was added, and the bottle was returned to the muffle furnace for further incineration over 1 h. The total ash is stated as a percentage of dry weight [5].

#### 2.3.8. Total Fat

Total fat was estimated using a previously defined method [5], following the Soxhlet method (Ankom XT15 Extractor, Ankom Technology, Macedon, NY, USA; Soxtec™).

#### 2.3.9. Total Fiber

The total fiber was estimated using an adapted version of a previously described method [23]. Briefly, 2 g of sample and 20 mL of deionized H_2_O were added, and the mixture was kept at 100 °C for 10 min. Then, 80 mL of 99% ethanol was added; the mixture was agitated and left in ice water for 35 min. The mixture was centrifuged using a Universal 320 R (Andreas Hettich GmbH & Co. KG, Tuttlingen, Germany) at 1500× *g* for 12 min at 4 °C; then, the filtrate was added to 100 mL of 85% ethanol, mixed, and centrifuged again. This step was repeated using 100 mL of 99% ethanol. Lastly, the filtrate was dehydrated at 80 °C for 24 h and assessed.

#### 2.3.10. Minerals

Date fruit, sweet potato, and jam samples were prepared for the determination of minerals, as defined previously [23]. The minerals were determined through inductively coupled plasma atomic emission spectrometry (Varian-Vista-MPX; Varian, Inc., Palo Alto, CA, USA), as outlined in the manufacturer’s manual.

### 2.4. Bioactive Compounds and Antioxidants

#### 2.4.1. Extraction of Carotenoids

Carotenoids in date, sweet potato, and jam samples (5 g) were liquified in a 45 mL hexane/acetone (60:40) solution. The mixture was homogenized with constant shaking for at least 15 min, with the addition of 5 mL of water. Then, a 750 W ultrasonic processor (VC 750, Sonics and Materials Inc., Newtown, CT, USA) was used for 17 min and at a temperature of 32 °C. The mixture was then filtered through a Whatman 150 mm, No. 42 filter paper, and the filtrate was composed and dried under N_2_. After complete drying, the residue was redissolved in 2 mL of the mobile phase containing acetonitrile, methanol, and ethyl acetate (730:200:70 mL) and filtered through a 0.45 µm syringe filter into vials, set for injection in a Waters ACQUITY UPLC with TUV. The injection volume was 10 µL, and separation was carried out in an ACQUITY UPLC BEH Amide Column C18 (2.1 × 50 mm, 1.7 µm), maintained at 30 °C with a run time of 10 min. The mobile phase was run at a flow rate of 0.2 mL/min. An ACQUITY UPLC PDA detector was used at 450 nm [24].

#### 2.4.2. Extraction of Phenols

To extract phenolic compounds for HPLC analysis, accelerated solvent extraction (ASE, 350, Dionex Co., Thermo Scientific, Carlsbad, CA, USA) was utilized. Stainless-steel extraction cells (11 mL) and amber collection vials (40 mL) were employed, along with ASE Prep DE. The extraction was conducted at a temperature of 25 °C and a pressure of 1500 psi, with a static time of 5 min, four static cycles, a flush of 75%, and a purge of 90 s, following the manufacturer’s instructions with some modifications. Acidified water (adjusted to pH 2 with HCl) was used for the extraction. The entire phenolic fraction was eluted with methanol (300 mL) and then reduced to dryness under reduced pressure (50 °C). The residue was resuspended in 5 mL of distilled water and extracted three times with 5 mL of diethyl ether. The ether extracts were combined, concentrated under nitrogen, and dissolved in 1 mL of a 50:50 (*v*/*v*) methanol/water solution. Lastly, all extracts were filtered using a 0.45 µm mesh and analyzed by HPLC.

#### 2.4.3. Total Phenolics

The total phenolics were evaluated by conducting spectrophotometric analysis using Folin–Ciocâlteu’s phenol reagent, as previously reported [25]. The standard curve for total phenolics was calculated using a standard solution (0–100 mg/mL) of gallic acid, and the total phenolic content is stated as mg of gallic acid equivalent (GAE) per 100 g of sample.

#### 2.4.4. Total Flavonoids

Total flavonoid content was assessed using a previously reported method [26]. An aliquot (250 μL) of the extract or standard solution was mixed with 75 μL of a 5% NaNO_2_ solution and 1.25 mL of H_2_O. After 6 min, 150 μL of a 10% AlCl_3_ solution was added. After 5 min, 1 M NaOH solution (0.5 mL) was added, and the total volume was brought to 2.5 mL with H_2_O. The absorbance of the blank was measured at a wavelength of 510 nm. The results are stated in terms of mg of rutin equivalent (RE) per 100 g of sample.

#### 2.4.5. DPPH^•^ Free Radical-Scavenging Assay

A DPPH^•^ free radical-scavenging assay was performed to determine the activity of the samples against 1,1-diphenyl-2-picrylhydrazyl radicals. The percentage inhibition of these radical compounds was calculated using the following equation:

% Inhibition DPPH^•^ = (Abs. control − Abs. sample)/Abs. control × 100, (1)

where Abs. control denotes the absorbance of the DPPH solution without the test sample. The absorbance was measured using a Varian CARY 50 Scan UV/Vis spectrophotometer with a Cary 50 Microplate Reader Accessory (Varian, Inc., Walnut Creek, CA, USA) [27].

#### 2.4.6. ABTS^•^ Free Radical-Scavenging Assay

The ABTS^•^ free radical-scavenging assay was conducted to evaluate the ability of the samples to scavenge ABTS^•^ radicals. The results were compared with a previously reported method [28], and the percentage inhibition of these radical compounds was determined using Equation (1), as described above.

#### 2.4.7. Ferric-Reducing/Antioxidant Power (FRAP) Assay

The samples were subjected to the ferric-reducing/antioxidant power (FRAP) assay, following a previously reported method [28]. The FRAP reagent was prepared by mixing 10 mM TPTZ solution in 40 mM HCl, 20 mM FeCl_3_, and 0.3 M acetate buffer (pH 3.6) in a 1:1:10 (*v*/*v*/*v*) proportion. Next, 1 mL of the tested sample was mixed with 2 mL of freshly prepared FRAP reagent and incubated for 30 min at 37 °C. The absorbance was measured at 593 nm using a Varian CARY 50 Scan UV/Vis spectrophotometer with a Cary 50 Microplate Reader Accessory (Varian, Inc., Walnut Creek, CA, USA). A blank was prepared using deionized water, and FeSO_4_ was used to create the calibration curve. The FRAP values are reported in terms of µmol of Fe(II).

#### 2.4.8. Superoxide Anion Radical-Quenching Assay

The superoxide radical (O_2_^•−^)-scavenging capacity was measured through the amount of purple-colored diformazan formed from the NBT reduction encouraged by a superoxide radical-scavenging capacity nonenzymatic NADH/PMS/O_2_ system, which was used to generate O_2_^•−^ following a procedure reported previously [29]. Optical density was read at 560 nm for 5 min, and the results are stated as the percentage of inhibition.

### 2.5. Sensory Evaluation

Ethical considerations: For this study, we obtained the approval of the Ethics Committee of the Deanship of Scientific Research, King Faisal University (Protocol KFU-REC-2022-SEP–ETHICS192), as well as the approval of trained sensory evaluators who participated in the study.

Sensory analysis was conducted with panels consisting of a panel leader and 20 panel members who evaluated differently prepared jam samples from the Date Palm Research Center of Excellence and the Food and Nutrition Department, College of Agricultural, and Food Sciences, King Faisal University, KSA. The sensory profile assessment of the jams was carried out by a semitrained panel utilizing a hedonic scale from 1 to 9, where appearance, color, odor, taste, and overall acceptability were evaluated as key characteristics, with 9 and 1 representing ‘like extremely’ and ‘dislike extremely’, respectively [30]. In addition, the prepared samples were compared with a sample of commercial date jam purchased from a local market, Al-Hasah, KSA. Samples were evaluated for taste, color, odor, and appearance.

Water was used to wash the mouths of participants twice in between assessments. All sessions of the evaluation process were conducted in a climate-controlled sensory analysis booth (i.e., 20 °C room temperature, positive air pressure) and under white, fluorescent lighting [5].

### 2.6. Statistical Analysis

All analyses were conducted in triplicate (*n* = 3). Statistical evaluations were carried out using SPSS for Windows (version 26; SPSS Inc., Chicago, IL, USA). The variances in mean values across samples were determined (*p* < 0.05) using a one-way analysis of variance (ANOVA). Separation of means was also completed using Tukey’s multiple range tests. Values for the mean ± standard deviation were also obtained.

## 3. Results and Discussion

### 3.1. Physicochemical Properties of Raw Materials

The results in Table 2 indicate that the date fruit had a lower moisture content, higher carbohydrate content, and higher total fiber content, compared to sweet potato. Sweet potato, on the other hand, had a higher moisture content, lower carbohydrate content, and lower total fiber content. The protein and fat contents were higher in sweet potato, compared to date fruit. The pH value of sweet potato was significantly higher than that of date fruit, indicating that sweet potato is more alkaline. Additionally, sweet potato had a lighter color compared to date fruit. Parallel findings have previously been described [12,21,31,32,33,34,35,36]. The inconsistencies detected between our results and other results published in the literature are likely due to differences in fertilizer, maturity, geographic origin, season, growing and storage conditions, soil type, disease, and analysis methods [37,38,39]. These results suggest that date fruit and sweet potato have different nutritional profiles and can provide complementary benefits when mixed together in a balanced diet.

The results provided in Table 3 indicate that date fruit is a rich source of macrominerals such as potassium, phosphorus, and magnesium, with significantly higher contents compared to sweet potato. Potassium is an essential mineral that plays a vital role in various body functions, including regulating blood pressure, maintaining fluid balance, and supporting muscle and nerve function. Phosphorus is also essential for healthy bones and teeth, while magnesium is involved in many biochemical reactions in the body, including energy production and protein synthesis. The higher contents of these minerals in date fruit make it a highly nutritious food. From the data, it is clear that the date fruit is rich in macrominerals and microminerals, except for its calcium content. Comparable conclusions have previously been stated [12,21,31,32,33,34,35,36,40]. Again, the inconsistencies detected between our results and other results published in the literature are likely due to differences in fertilizer, maturity, geographic origin, season, growing and storage conditions, soil type, disease, and analysis methods [37,38,39].

### 3.2. Raw Material Bioactive Compounds and Antioxidant Activity

Phenolic and flavonoid compounds are the major antioxidants found in fruits and vegetables [41,42,43,44,45]. The bioactive compounds present in and antioxidant activity of the tested date fruit and sweet potato are presented in Table 4. The total phenolics in date fruit and sweet potato were 387.54 ± 2.23 and 187.67 ± 0.13 mg of gallic acid equivalent (GAE) per 100 g, respectively, while total flavonoids were 74.46 ± 0.71 and 68.57 ± 0.24 mg of rutin equivalent per 100 g, respectively. Thus, the date fruit had higher total phenolic and flavonoid contents than the sweet potato.

Moreover, the phenolic profile of date fruit regarding gallic, syringic, coumaric, caffeic, and ferulic acid, as well as catechin, epicatechin, and β-carotene, was 16.22 ± 0.26, 9.39 ± 0.17, 6.74 ± 0.21, 13.96 ± 0.52, 18.02 ± 0.37, 4.89 ± 0.34, 6.56 ± 0.19, and 0.39 ± 0.02 mg/100 g fresh weight, respectively; in sweet potato, the phenolic profile regarding gallic, coumaric, caffeic, and ferulic acid, as well as epicatechin and β-carotene, was 2.08 ± 0.14, 1.39 ± 0.41, 1.48 ± 0.36, 28.06 ± 0.72, 0.34 ± 0.01, and 518.94 ± 0.64 mg/100 g fresh weight, respectively. Syringic acid and catechin were not detected in sweet potato, and the levels of phenolic contents were lower than in date fruit, except for ferulic acid and β-carotene.

The antioxidant activities of date fruit and sweet potato, in terms of FRAP (mmol ferrous equivalents/100 g), DPPH, ABTS, and superoxide percentage inhibition, are detailed in Table 4. In date fruit, the FRAP value was 907.12 ± 2.74 ferrous equivalents/100 g fresh weight, lower than that in sweet potato (1024.64 ± 6.24 mmol). Nevertheless, the percentage inhibition of DPPH, ABTS, and superoxide for date fruit was 5.27% ± 0.42%, 67.51% ± 1.08%, and 19.19% ± 0.74%, respectively, while that for sweet potato was 78.31% ± 0.75%, 82.47% ± 2.36%, and 42.14 ± 0.86%, respectively. The higher values in sweet potato were due to the higher content of β-carotene than in date fruit, exhibiting radical-scavenging capacity from DPPH, ABTS, and superoxide. A comparison with previous studies revealed that the differences in antioxidant activity in our data were typically in the same range, with some exceptions [12,21,31,32,33,34,35,36,40]. Variations in phenolic compound structures and antioxidant activity can be influenced by the fruit variety, climatic conditions, geography, soil, fertilization, degree of ripeness, cultivation practices, and extraction method [46,47,48,49]. The variations in the results for antioxidant activity may have been due to several factors, e.g., the genetic potential of individual species for polyphenol biosynthesis. In addition to the genetic (varietal) factors, the ripening stage may be critical [50].

### 3.3. Physicochemical Properties of Jams

Jams were made with different proportions of date fruit and sweet potato (DP1–DP4), as detailed in Table 5. We found that, as the proportion of sweet potato increased, the moisture content also increased, while the total fiber content decreased. DP1 had the highest carbohydrate content at 71.83% ± 0.40%, while DP3 had the lowest carbohydrate content at 65.93% ± 0.24%. DP3 had the lowest protein content at 0.62% ± 0.01%, while DP1 had the highest protein content at 0.96% ± 0.02%. The fat content varied slightly among the different jams, with DP2 having the lowest fat content at 0.16% ± 0.02%, but DP1 having the highest fat content at 0.15% ± 0.01%. The ash content varied among the different jams, with DP3 having the lowest ash content at 1.03% ± 0.03%, but DP1 having the highest ash content at 1.41% ± 0.03%. The pH values were similar among the different jams, ranging from 3.61 ± 0.05 to 3.98 ± 0.03. These results corroborate previous findings in other fruit jams [51,52,53,54]. The results indicate that the proportion of sweet potatoes used in the jam affects the nutritional composition of the final product. Jams made with a higher proportion of sweet potato had a higher moisture content, lower total fiber content, and lower protein content, while the carbohydrate and ash contents varied among the different jams. The results suggest that the proportion of sweet potatoes used in the jam-making process can be adjusted to achieve the desired nutritional composition. Additionally, the pH values of all the jams were acidic, which is important for the preservation of the jam.

Overall, the results of this study demonstrate that mixing sweet potato with date fruit is a viable option for making jams.

### 3.4. Color Parameters of Formulated Jams

**Figure 2:**
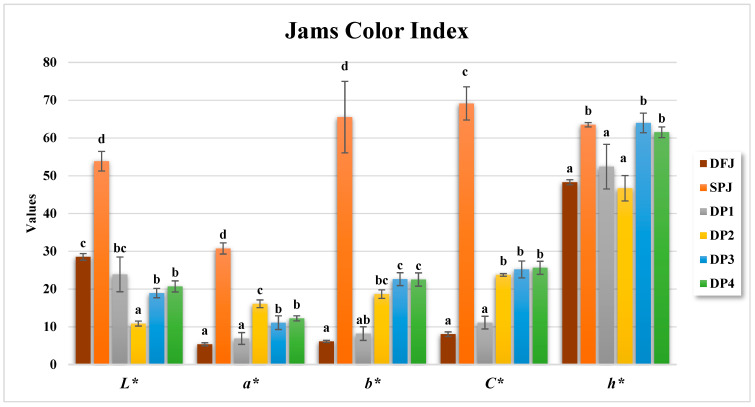
Color parameters for the formulated jams. Data are presented as the mean ± standard deviation (*n* = 3). Values with different letters indicate a significant difference according to Tukey’s test (*p* ≤ 0.05).

**Figure 3:**
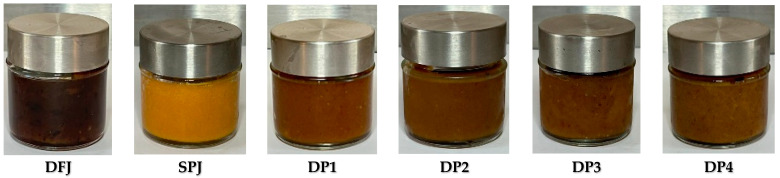
The visual appearance of the formulated jams incorporating SPJ and DFJ at different ratios.

Color is one of the most significant quality considerations that affects consumers in judging the acceptability and overall quality of jam. The color properties of the jams were analyzed, and the results are presented in Figure 2 and Figure 3. The L* value of DFJ was 28.50 ± 0.88, while SPJ had a significantly (*p* ≤ 0.05) higher L* value of 53.84 ± 2.61. Among the jams made with different proportions of date fruit and sweet potato, DP2 had the lowest L* value at 10.85 ± 0.66, while DP1 had the highest L* value at 23.86 ± 4.60. The a* value of DFJ was 5.35 ± 0.40, while SPJ had a significantly (*p* ≤ 0.05) higher a* value of 30.73 ± 1.48. Among the jams made with different proportions of date fruit and sweet potato, DP2 had the highest a* value at 16.07 ± 1.02, while DP1 had the lowest a* value at 6.89 ± 1.56. The b* value of DFJ was 6.12 ± 0.29, while SPJ had a significantly (*p* ≤ 0.05) higher b* value of 65.52 ± 9.47. Among the jams made with different proportions of date fruit and sweet potato, DP3 had the highest b* value at 22.6 ± 1.70, while DP4 had the lowest b* value at 22.51 ± 1.76.

Overall, the results showed that the sweet potato-based jam (SPJ) had significantly higher L*, a*, and b* values than the date fruit-based jam (DFJ) and jams made with different proportions of date fruit and sweet potato (DP1–DP4). The higher L* value of SPJ indicated a lighter color, while the lower L* values of DFJ and DP1–DP4 indicated darker colors. The higher a* value of SPJ indicated a more intense red color, while the lower a* values of DFJ and DP1–DP4 indicated a less intense red color. Lastly, the higher b* value of SPJ indicated a more intense yellow color, while the lower b* values of DFJ and DP1–DP4 indicated a less intense yellow color. The results also demonstrate that the samples vary in their lightness (L*) values, with some samples presenting darker (e.g., DP2 and DP3) and others presenting lighter (DFJ and SPJ). Similarly, the chroma values also vary between samples, with some samples having higher chroma values (SPJ) and others having lower chroma values (DP1).

The hue angle (°h) values specify the path of the color shift in each sample. Some samples demonstrate a shift near green (DP2) or yellow (DP4), while others demonstrate a shift in the direction of blue (SPJ). The hue angle values can help in defining the nature of the color difference between two samples.

It is important to note that the color properties of jams can also be influenced by factors such as the ripeness of the fruit, pH, and processing methods such as nonenzymatic browning, mostly caused by the caramelization of sugar and Maillard reactions [55].

Overall, the results highlight the differences in color properties between jams made with date fruit and sweet potato. These differences could influence consumer acceptance and preferences for these products. Further studies may explore the impact of color properties on consumer acceptance and preference for jams made with date fruit and sweet potato.

### 3.5. Mineral Content of Jams

**Figure 4:**
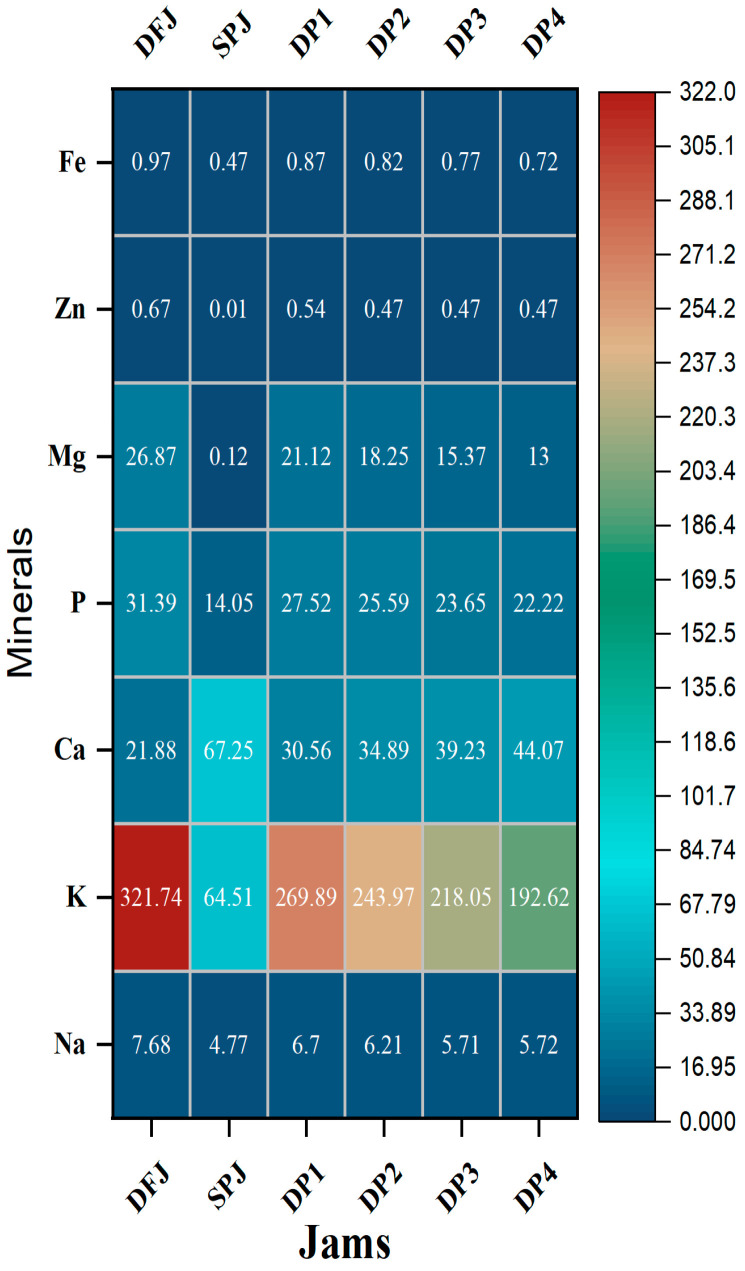
Heatmap showing the relative contents of minerals in the jams (*n* = 3). The results are reported on a wet basis.

The mineral contents of the formulated jam samples are presented in Figure 4. The mineral profile indicated the presence of beneficial mineral elements, such as the macrominerals sodium (Na), potassium (K), calcium (Ca), phosphorus (P), and magnesium (Mg), as well as the microminerals zinc (Zn) and iron (Fe), which varied significantly (*p* ≤ 0.05) in the date fruit and sweet potato formulations: 4.77 ± 0.10 to 7.68 ± 0.17, 321.74 ± 0.90 to 64.51 ± 0.17, 21.88 ± 0.13 to 67.25 ± 0.18, 14.05 ± 0.18 to 31.39 ± 0.32, and 0.12 ± 0.01 to 26.87 ± 0.14 mg/100 g jam, respectively, for the macrominerals in formulated jams, and 0.01 ± 0.00 to 0.67 ± 0.01 and 0.47 ± 0.03 to 0.97 ± 0.03 mg/100 g jam, respectively, for the microminerals. The lowest values of minerals were presented in SPJ, while the highest values were in DFJ (except for calcium content, which was the highest in SPJ). Mineral contents were found to decrease slightly but significantly (*p* ≤ 0.05) in DP1–DP4 compared to DFJ and SPJ. Likewise, the results for jams comprising a mixture of date fruit and sweet potato demonstrate that they are good sources of minerals and are within the recommended values for healthy living [18].

### 3.6. Bioactive Compounds in Formulated Jams

Table 6 shows the total phenols, total flavonoids, and phenolic profile for DFJ, SPJ, and the mixed jams DP1–DP4 with different proportions of DFJ and SPJ. The raw materials of the date fruit and sweet potato had higher phenolic content than the developed jams. It was also noted that the heat treatment used to produce the jams slightly decreased the phenolic content of date fruit and sweet potato. Interestingly, the 100% date fruit and sweet potato jams showed higher total phenol content than the mixed jams: 232.74 ± 1.25 and 114.05 ± 1.87 mg GAE/100 g, respectively, while DP1–DP4 ranged from 208.60 ± 1.37 to 172.89 ± 1.56 mg GAE/100 g. The same pattern was observed in the total flavonoid content.

Moreover, the phenolic profile of DFJ regarding gallic, syringic, coumaric, caffeic, and ferulic acid, as well as catechin, epicatechin, and β-carotene, was 8.90 ± 0.16, 5.07 ± 0.13, 3.64 ± 0.12, 7.81 ± 0.18, 10.61 ± 0.37, 2.40 ± 0.39, 4.54 ± 0.21, and 0.17 ± 0.00 mg/100 g jam, respectively; in SPJ, the phenolic profile regarding gallic, coumaric, caffeic, and ferulic acid, as well as epicatechin and β-carotene, was 1.15 ± 0.07, 0.90 ± 0.03, 0.83 ± 0.05, 15.33 ± 0.36, 0.18 ± 0.00, and 310.77 ± 1.68 mg/100 g jam, respectively. Syringic acid and catechin were not detected in SPJ, and the levels of phenolics in SPJ were lower than in DFJ except for ferulic acid and β-carotene. The phenolic profile in DP1–DP4 jams was significantly (*p* ≤ 0.05) lower than in DFJ, but increased significantly (*p* ≤ 0.05) compared to SPJ. This indicates that the mixing of DFJ and SPJ significantly increased (*p* ≤ 0.05) the nutritional value of DP1–DP4.

### 3.7. Antioxidant Activity of Jams

**Figure 5:**
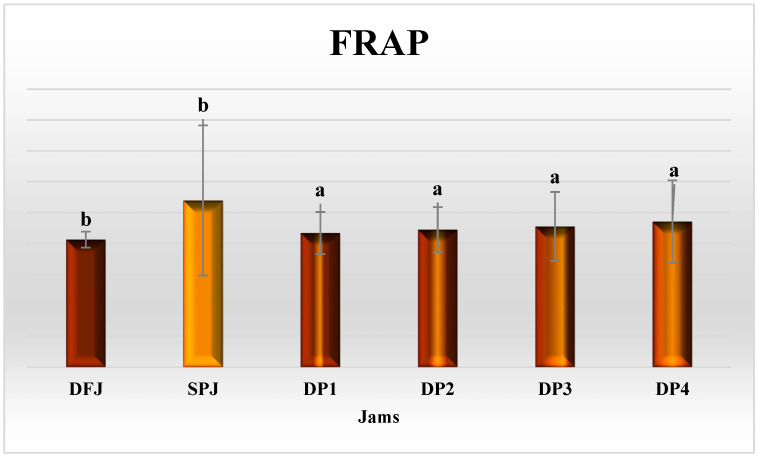
Ferric-reducing antioxidant power (FRAP) activity in jams (*n* = 3). The results are reported on a wet basis. Values with different superscript letters indicate a significant difference according to Tukey’s test (*p* ≤ 0.05).

**Figure 6:**
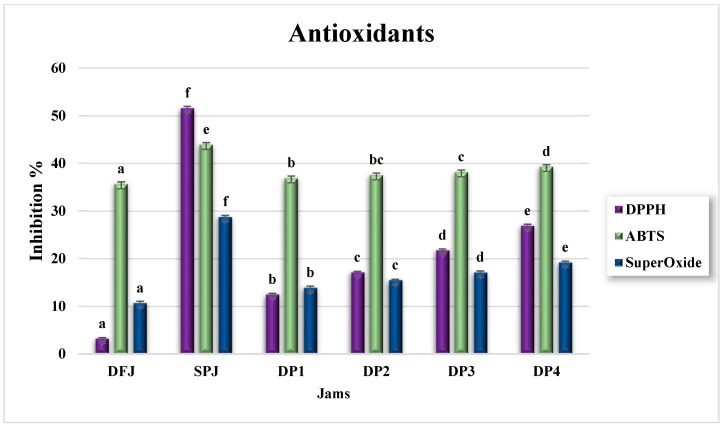
Antioxidant activity of jam, with respect to DPPH, ABTS, and superoxide (*n* = 3). The results are reported on a wet basis. Values with different superscript letters indicate a significant difference according to Tukey’s test (*p* ≤ 0.05).

The antioxidant activity of DFJ and SPJ, in terms of FRAP (mmol ferrous equivalents/100 g), DPPH, ABTS, and superoxide percentage inhibition is illustrated in Figure 5 and Figure 6. In DFJ, the value of FRAP (601.28 ± 2.59) was insignificantly (*p* ≥ 0.05) lower than that in SPJ (613.28 ± 24.31 mmol ferrous equivalents/100 g jam). The mixed jams DP1–DP4 presented significantly reduced (*p* ≤ 0.05) values, compared to DFJ and SPJ, but with insignificant (*p* ≥ 0.05) variation among them.

Nevertheless, the percentage inhibition of DPPH, ABTS, and superoxide for DFJ was 3.19% ± 0.23%, 35.66% ± 0.40%, and 10.67% ± 0.40%, respectively, while that for SPJ was 51.58% ± 0.40%, 43.93% ± 0.46%, and 28.74 ± 0.31%, respectively. The higher percentage inhibition of SPJ was considered to be due to its higher content of β-carotene than DFJ, which has the ability to scavenge DPPH, ABTS, and superoxide.

On the other hand, the mixed jams DP1–DP4 showed significantly reduced (*p* ≤ 0.05) values, compared to DFJ and SPJ, but they increased significantly (*p* ≤ 0.05) on a gradual basis with an increase in the percentage mixing with SPJ, due to the increasing percentage of β-carotene.

According to a comparison with previous studies, our data showed a similar range of antioxidant activity [12,21,31,32,33,34,35,36,40]. However, there were some variations in the results. These discrepancies may have been due to differences in phenolic compound structures and antioxidant activity, which can be influenced by various factors such as fruit variety, climatic conditions, geography, soil, fertilization, degree of ripeness, cultivation practices, and extraction methods [46,47,48,49]. The differences in antioxidant activity could also be attributed to the genetic potential of individual species for polyphenol biosynthesis. Furthermore, the ripening stage of the fruit may also play a critical role [50].

### 3.8. Correlation Analyses

**Figure 7:**
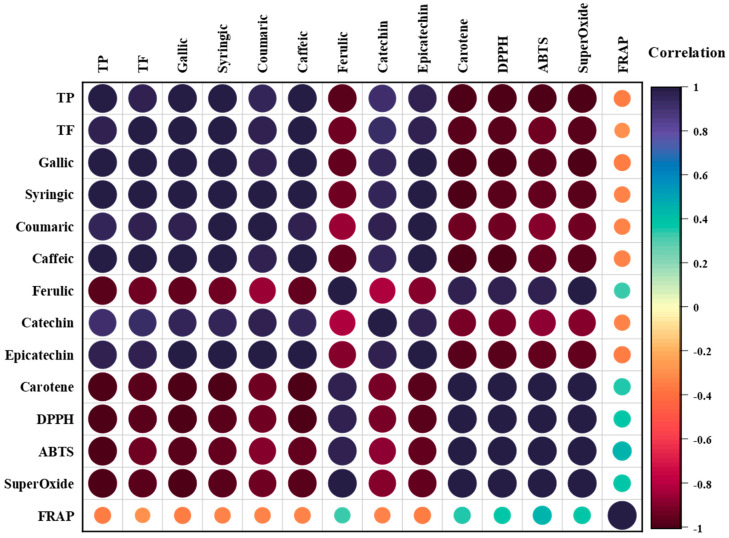
Correlation heatmap among the contents of TP, TF, phenolic compounds, and antioxidant activity in jams, evaluated using a color diagram.

The Pearson correlations were calculated in order to understand the impact of the phenolic compounds and flavonoids on the antioxidant effects of the formulated jams. In this situation, a positive correlation would be the necessary conclusion. Figure 7 represents the Pearson correlations among the results for TPC, TFC, phenolic compounds, and antioxidant activities, evaluated as a correlation heatmap.

As illustrated in Figure 7, the TP analysis accomplished very strong positive correlations with TF (*r*^2^ = 0.978), gallic acid (*r*^2^ = 0.992), syringic acid (*r*^2^ = 0.982), coumaric acid (*r*^2^ = 0.941), caffeic acid (*r*^2^ = 0.991), catechin (*r*^2^ = 0.912), and epicatechin (*r*^2^ = 0.971). These results indicated that an increase in TP led to enhanced antioxidant responses, with the polyphenols from the jams as the main bioactive compounds contributing to the antioxidant activities of the functional jams. In contrast, strong negative correlations with ferulic acid (*r*^2^ = −0.973), β-carotene (*r*^2^ = −0.999), DPPH (*r*^2^ = −0.999), ABTS (*r*^2^ = −0.981), and superoxide (*r*^2^ = −0.997) were observed, while TP had a weak negative correlation with FRAP (*r*^2^ = −0.344). These results indicate that a higher scavenging activity against this reactive species was accomplished by TP, such as in functional jams. Furthermore, for TF, gallic acid, syringic acid, coumaric acid, caffeic acid, catechin, and epicatechin had the same strong positive and strong negative trends as TP. However, while ferulic acid had a strong negative correlation with TP, for TF, negative correlations were observed for gallic, syringic, coumaric, and caffeic acids, as well as catechin and epicatechin (*r*^2^ = −0.973, −0.938, −0.941, −0.921, −0.855, −0.940, −0.816, and −0.898, respectively), while strong positive correlations with β-carotene, DPPH, ABTS, and superoxide (*r*^2^ = 0.974, 0.976, 0.975, and 0.983, respectively) were observed. Furthermore, TP also had a weak positive correlation with FRAP (*r*^2^ = 0.324). Nevertheless, FRAP only showed weak positive correlations with ferulic acid (*r*^2^ = 0.324), β-carotene (*r*^2^ = 0.359), DPPH (*r*^2^ = 0.367), ABTS (*r*^2^ = 0.443), and superoxide (*r*^2^ = 0.379), but weak negative correlations with TP (*r*^2^ = −0.344), TF (*r*^2^ = −0.258), gallic acid (*r*^2^ = −0.348), syringic acid (*r*^2^ = −0.327), coumaric acid (*r*^2^ = −0.326), caffeic acid (*r*^2^ = −0.336), catechin (*r*^2^ = −0.322), and epicatechin (*r*^2^ = −0.356).

### 3.9. Sensory Evaluation

Sensory challenges were conducted to evaluate the qualities of a food product with respect to the five senses (smell, hearing, sight, touch, and taste), facilitating the consumer-oriented creation of new functional products. The nine-point hedonic scale is an operative and simple unconditional measurement tool that has been extensively used to discover sensory differences among foods and beverages; it is regularly used to predict the acceptance of novel functional food products by potential consumers.

**Figure 8:**
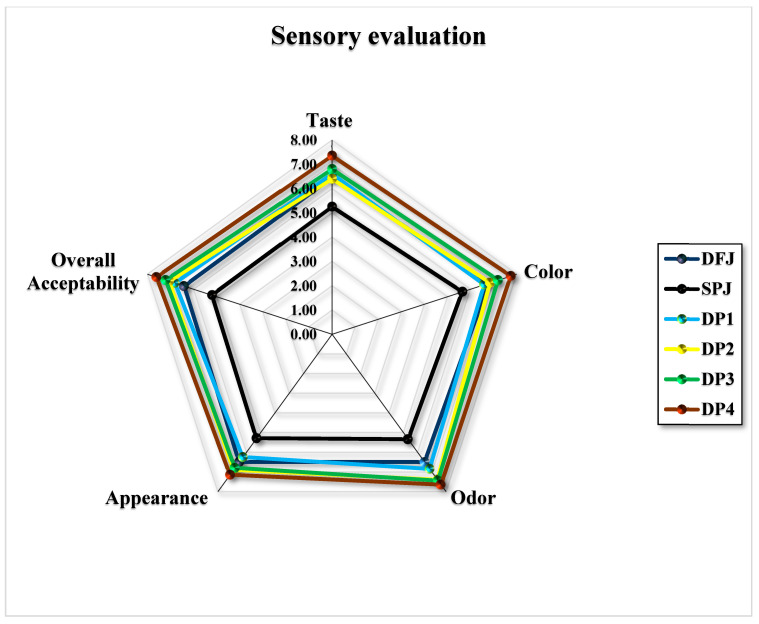
Radar plot describing sensory evaluations of jams formulated by mixing date fruit and sweet potato (*n* = 20).

Figure 8 illustrates the sensory analysis, which involved preference tests regarding the taste, color, odor, appearance, and overall acceptability. The SPJ containing 100% sweet potato had the lowest score in all preferences tested (5.25 ± 0.79, 5.65 ± 0.81, 5.35 ± 0.81, 5.30 ± 0.92, and 5.20 ± 0.77 for taste, color, odor, appearance, and overall acceptability, respectively), followed by DFJ, while the mixed formulations of the jams increased in score gradually with an increased percentage of mixing. DP4, which had 50% DFJ and 50% SPJ, presented the best product scores in all tested sensory preferences (7.35 ± 1.53, 7.75 ± 091, 7.65 ± 0.99, 7.15 ± 1.18, and 7.60 ± 1.23, respectively).

### 3.10. Correlation Analysis of Color Results Determined Instrumentally and Evaluated Sensorily

**Figure 9:**
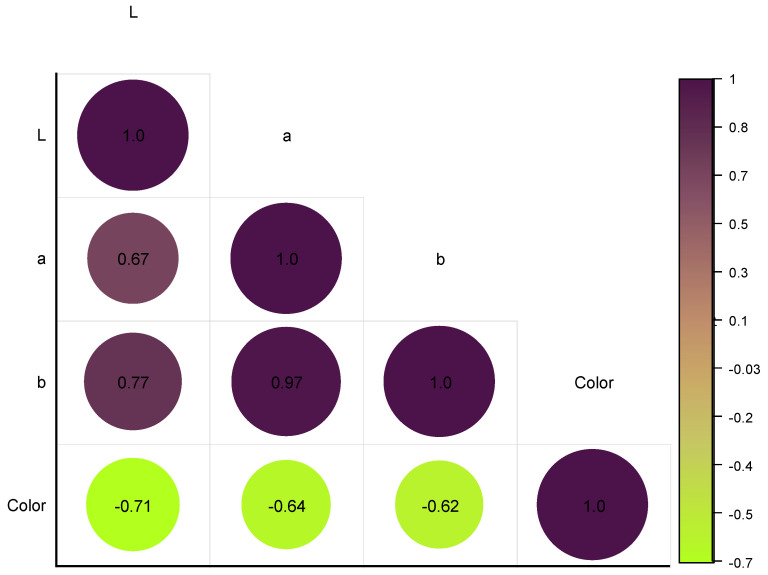
Heatmap graph depicting the color results determined instrumentally and evaluated sensorily in jams, using a color diagram.

The results in Figure 9 show that there was a strong positive correlation between L* and a* values (*r* = 0.676) and a very strong positive correlation between a* and b* values (*r* = 0.967 **). There was also a strong positive correlation between L* and b* values (*r* = 0.780). These results suggest that, as the L* value increased (i.e., the color became lighter), the a* and b* values also tended to increase (i.e., the color became more red and yellow, respectively). Additionally, as the a* value increased (i.e., the color became redder), the b* value also tended to increase (i.e., the color became more yellow). These relationships are commonly observed in color science, and they are known as opponent process theory.

The results also indicated a strong negative correlation between the L* value and the sensory evaluation of color (*r* = −0.781), indicating that, as the L* value increased (i.e., the color became lighter), the sensory evaluation of color tended to decrease (i.e., the color was perceived as less intense). There was also a moderate negative correlation between the a* value and sensory evaluation of color (*r* = −0.643), as well as a weak negative correlation between the b* value and sensory evaluation of color (*r* = −0.623), indicating that, as the a* and b* values increased (i.e., the color became more red and yellow, respectively), the sensory evaluation of color tended to decrease (i.e., the color was perceived as less intense).

### 3.11. Correlation Analysis of Color Parameter and Antioxidant Activity

**Figure 10:**
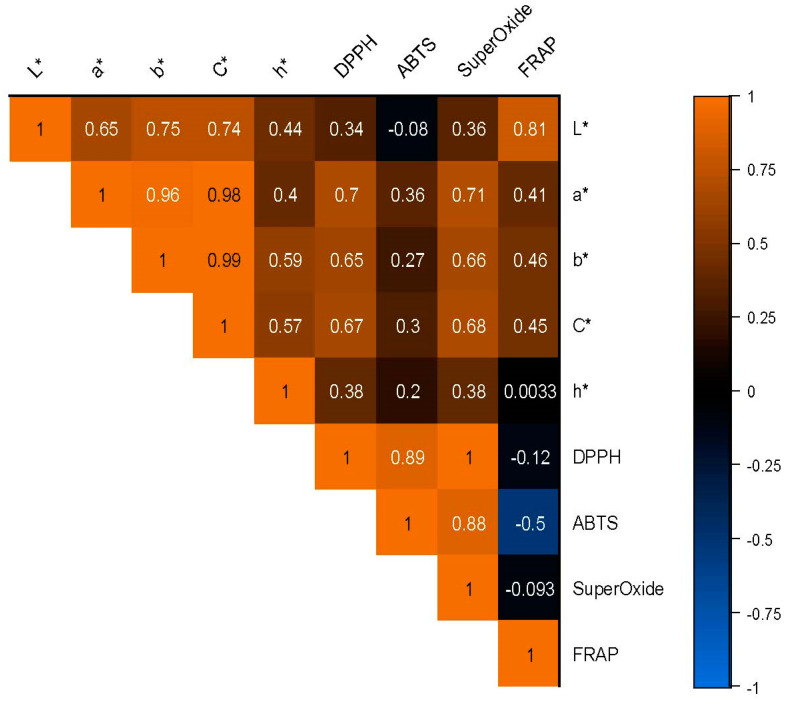
Heatmap graph depicting the color parameters and antioxidant activity evaluated using a color diagram.

Figure 10 illustrates that L* is significantly correlated with a* (*r* = 0.652 **) and b* (*r* = 0.749 **), indicating that the lightness of a substance is related to its redness and yellowness. C* is also significantly correlated with a* (*r* = 0.975 **) and b* (*r* = 0.994 **), suggesting that the chroma of a substance is strongly related to its colorfulness. Furthermore, the DPPH is strongly correlated with ABTS (*r* = 0.893 **) and superoxide (*r* = 0.999 **), indicating that these assays are highly related and may be used interchangeably. However, it should be noted that ABTS is negatively correlated with superoxide (*r* = −0.502 *), suggesting that these assays may not always afford identical results. Remarkably, the hue angle of a substance is only weakly correlated with the other variables. Lastly, the FRAP is strongly correlated with L* (*r* = 0.811 **), a* (*r* = 0.414), b* (*r* = 0.462), and C* (*r* = 0.454). This suggests that color and antioxidant capacity may be related in complex ways and that different colorimetric variables may be more or less important depending on the specific test being used.

In conclusion, the results of this study provide valuable information about the relationships between colorimetric variables and antioxidant capacity. While some variables are strongly correlated, others are only weakly related, suggesting that different aspects of color perception may be more or less important for determining antioxidant capacity depending on the specific assay being used. Further research is needed to confirm these findings and better understand the complex relationships between color and antioxidant capacity.

## 4. Conclusions

The results confirmed the potential of using second-grade date fruit and nonstandard sweet potato tubers as raw materials for producing novel functional jams with high nutritional and antioxidant value. The optimal ratio of date fruit paste and sweet potato paste was found to be 50:50, which resulted in the highest overall acceptability among the sensory panelists. The mixed fruit jam also showed a high content of bioactive compounds, including phenolics, flavonoids, and β-carotene, which have various health benefits. The results of this study suggest that valorizing the byproducts of date fruit and sweet potato can provide a sustainable and economical way of developing value-added products that can enhance food security and diversity in the region. Therefore, it is recommended that further research be conducted to optimize the processing conditions and shelf-life of the mixed fruit jam, as well as evaluate its impact on human health and consumer preference.

## Figures and Tables

**Table 1:** Ingredients and experimental design of date fruit and sweet potato tuber pastes per 100 g of combined paste to determine the optimal jam treatment.

Ingredients/100 g Jam	Khalas Date Fruit				Control Treatments	
	DP_1_	DP_2_	DP_3_	DP_4_	DFJ	SPJ
Date fruit	40%	35%	30%	25%	50%	0.0%
Sweet potato	10%	15%	20%	25%	0.0%	50%
White sugar, sucrose	11%	13.5%	16.5%	17.5%	20%	65%
Citric acid	0.030%	0.030%	0.030%	0.030%	0.030%	0.030%
Ascorbic acid	0.035%	0.035%	0.035%	0.035%	0.035%	0.035%
Sterile distilled water	50%	50%	50%	50%	50%	50%
Total water loss	11%	13.5%	16.5%	17.5%	20%	65%
Total	100.065	100.065	100.065	100.065	100.065	100.065

**Table 2:** The physicochemical properties and color of date fruit and sweet potato [20,21].

Contents	Date Fruit	Sweet Potato
	(g/100 g wt.)	
Moisture %	22.07 ± 0.18	71.17 ± 0.22
TS %	77.93 ± 1.01	28.83 ± 0.37
Carbohydrate %	66.51 ± 0.28	20.57 ± 0.16
Protein %	2.35 ± 0.28	3.64 ± 0.24
Fat %	0.16 ± 0.02	0.36 ± 0.01
Total fiber %	7.35 ± 0.40	2.81 ± 0.73
Ash %	1.56 ± 0.11	1.44 ± 0.41
pH	5.83 ± 0.07	7.17 ± 0.05
a*	13.75 ± 0.13	−1.08 ± 0.81
b*	20.23 ± 0.11	18.54 ± 0.99
L*	26.57 ± 0.08	79.17 ± 1.35
°h	55.70 ± 027	93.30 ± 2.04
C*	24.50 ± 0.78	18.50 ± 0.69

Data are presented as the mean ± standard deviation (*n* = 3). L*, lightness; a*, redness; b*, yellowness, °h, hue angle, C*, chroma.

**Table 3:** Macromineral and micromineral contents in the raw material of date fruit and sweet potato.

Minerals	Date Fruit	Sweet Potato
	(mg/100 g)	
Na	8.71 ± 0.31	5.33 ± 0.41
K	402.32 ± 3.22	76.34 ± 1.02
Ca	39.82 ± 0.68	84.23 ± 0.83
P	57.37 ± 0.74	27.18 ± 0.48
Mg	53.23 ± 0.48	0.18 ± 0.03
Zn	1.43 ± 0.08	0.07 ± 0.01
Fe	1.84 ± 0.12	0.95 ± 0.02

Data are presented as the mean ± standard deviation (*n* = 3). The results are reported on a wet basis.

**Table 4:** Bioactive compounds and antioxidant activity of raw material.

Contents	Date Fruit	Sweet Potato
	(mg/100 g)	
Total phenolics (mg of gallic acid equivalent (GAE)/100 g)	387.54 ± 2.23	187.67 ± 0.13
Total flavonoids (mg of rutin equivalent/100 g)	74.46 ± 0.71	68.57 ± 0.24
Gallic acid	16.22 ± 0.26	2.08 ± 0.14
Syringic acid	9.39 ± 0.17	ND
Coumaric acid	6.74 ± 0.21	1.39 ± 0.41
Caffeic acid	13.96 ± 0.52	1.48 ±0.36
Ferulic acid	18.02 ± 0.37	28.06 ±0.72
Catechin	4.89 ± 0.34	ND
Epicatechin	6.56 ± 0.19	0.34 ± 0.01
*β*-Carotene	0.39 ± 0.02	518.94 ± 0.64
FRAP (mmol ferrous equivalents/100 g)	907.12 ± 2.74	1024.64 ± 6.24
DPPH (inhibition %)	5.27 ± 0.42	78.31 ± 0.75
ABTS (inhibition %)	67.51 ± 1.08	82.47 ± 2.36
Superoxide (inhibition %)	19.19 ± 0.74	42.14 ± 0.86

Data are presented as the mean ± standard deviation (*N* = 3). ND, not detected. The results are reported on a wet basis.

**Table 5:** Physicochemical properties of jams.

Physicochemical Properties of Jams									
Jams	Moisture %	TS %	TSS (°Brix) %	Carbohydrate %	Protein %	Fat %	Total Fiber %	Ash %	pH
DFJ 100%	25.89 ± 3.52 ^a^	74.11 ± 3.52 ^b^	64.00 ± 0.70 ^b^	67.00 ± 0.26 ^c^	1.30 ± 0.03 ^d^	0.12 ± 0.02 ^a^	4.62 ± 0.20 ^e^	1.78 ± 0.04 ^d^	3.19 ± 0.04 ^a^
SPJ 100%	28.87 ± 0.18 ^ab^	71.13 ± 0.18 ^ab^	66.00 ± 0.27 ^c^	69.00 ± 0.17 ^d^	1.59 ± 0.40 ^e^	0.23 ± 0.02 ^c^	1.62 ± 0.05 ^a^	1.91 ± 0.03 ^e^	3.18 ± 0.08 ^a^
DP1 80:20	33.16 ± 1.58 ^b^	65.84 ± 1.58 ^a^	64.00 ± 0.22 ^b^	71.83 ± 0.40 ^e^	0.96 ± 0.02 ^c^	0.15 ± 0.01 ^ab^	3.62 ± 0.17 ^d^	1.41 ± 0.03 ^c^	3.61 ± 0.05 ^b^
DP2 70:30	31.46 ± 0.88 ^b^	68.54 ± 0.88 ^a^	62.00 ± 0.41 ^a^	68.53 ± 0.19 ^d^	0.79 ± 0.03 ^b^	0.16 ± 0.02 ^ab^	3.12 ± 0.15 ^c^	1.22 ± 0.02 ^b^	3.77 ± 0.06 ^c^
DP3 60:40	32.77 ± 2.84 ^b^	67.23 ± 2.84 ^a^	63.00 ± 0.19 ^ab^	65.93 ± 0.24 ^b^	0.62 ± 0.01 ^a^	0.17 ± 0.01 ^b^	2.62 ± 0.13 ^b^	1.03 ± 0.03 ^a^	3.96 ± 0.04 ^d^
DP4 50:50	34.34 ± 3.52 ^b^	65.66 ± 0.78 ^a^	63.00 ± 0.50 ^ab^	60.63 ± 0.71 ^a^	0.95 ± 0.01 ^c^	0.18 ± 0.01 ^b^	2.62 ± 0.12 ^b^	1.35 ± 0.03 ^c^	3.98 ± 0.03 ^d^

Data are presented as the mean ± standard deviation (*n* = 3). Values with the different superscript letters within the same column indicate a significant difference according to Tukey’s test (*p* ≤ 0.05). The results are reported on a wet basis.

**Table 6:** Bioactive compounds in formulated jams.

Bioactive Compounds mg/100 g of Jams										
Jams	TPC	TFC	Gallic Acid	Syringic Acid	Coumaric Acid	Caffeic Acid	Ferulic Acid	Catechin	Epicatechin	*β*-Carotene
DFJ 100%	232.74 ± 1.25 ^f^	44.68 ± 0.48 ^e^	8.90 ± 0.16 ^f^	5.07 ± 0.13 ^e^	3.64 ± 0.12 ^e^	7.81 ± 0.18 ^f^	10.61 ± 0.37 ^a^	2.40 ± 0.39 ^d^	4.54 ± 0.21 ^e^	0.17 ± 0.00 ^a^
SPJ 100%	114.05 ± 1.87 ^a^	38.80 ± 0.45 ^a^	1.15 ± 0.07 ^a^	ND ^a^	0.90 ± 0.03 ^a^	0.83 ± 0.05 ^a^	15.33 ± 0.36 ^d^	ND ^a^	0.18 ± 0.00 ^a^	310.77 ± 1.68 ^f^
DP1 80:20	208.60 ± 1.37 ^e^	43.11 ± 0.32 ^d^	6.95 ± 0.11 ^e^	3.66 ± 0.10 ^d^	2.69 ± 0.09 ^d^	6.01 ± 0.14 ^e^	11.15 ± 0.23 ^ab^	1.77 ± 0.19 ^c^	3.27 ± 0.17 ^d^	61.89 ± 0.34 ^b^
DP2 70:30	196.53 ± 1.43 ^d^	42.32 ± 0.25 ^cd^	5.98 ± 0.09 ^d^	2.95 ± 0.09 ^c^	2.22 ± 0.08 ^c^	5.11 ± 0.11 ^d^	11.42 ± 0.16 ^b^	1.08 ± 0.27 ^b^	2.63 ± 0.15 ^c^	92.75 ± 0.50 ^c^
DP3 60:40	184.46 ± 1.49 ^c^	41.53 ± 0.19 ^bc^	5.00 ± 0.07 ^c^	2.24 ± 0.08 ^b^	1.74 ± 0.07 ^b^	4.22 ± 0.09 ^c^	11.69 ± 0.09 ^b^	0.77 ± 0.10 ^b^	2.00 ± 0.12 ^b^	123.61 ± 0.67 ^d^
DP4 50:50	172.89 ± 1.56 ^b^	41.24 ± 0.16 ^b^	4.53 ± 0.04 ^b^	2.04 ± 0.07 ^b^	1.77 ± 0.05 ^b^	3.82 ± 0.07 ^b^	12.47 ± 0.05 ^c^	0.80 ± 0.08 ^b^	1.86 ± 0.10 ^b^	154.97 ± 0.84 ^e^

Data are presented as the mean ± standard deviation (*n* = 3). Values with different superscript letters within the same column indicate a significant difference according to Tukey’s test (*p* ≤ 0.05). TPC, total phenolic content (mg of GAE/100 g); TFC, total flavonoid content (mg of rutin equivalent/100 g). The results are reported on a wet basis.

## Data Availability

Data is contained within the article.

## References

[1] Kandemir K., Piskin E., Xiao J., Tomas M., Capanoglu E. (2022). Fruit Juice Industry Wastes as a Source of Bioactives. J. Agric. Food Chem..

[2] Rao M., Bast A., de Boer A. (2021). Valorized Food Processing By-Products in the EU: Finding the Balance between Safety, Nutrition, and Sustainability. Sustainability.

[3] Caponio F., Piga A., Poiana M. (2022). Valorization of Food Processing By-Products. Foods.

[4] Iriondo-DeHond M., Miguel E., del Castillo M. (2018). Food Byproducts as Sustainable Ingredients for Innovative and Healthy Dairy Foods. Nutrients.

[5] Alqahtani N.K., Alnemr T.M., Alqattan A.M., Aleid S.M., Habib H.M. (2023). Physicochemical and Sensory Properties and Shelf Life of Block-Type Processed Cheeses Fortified with Date Seeds (*Phoenix dactylifera* L.) as a Functional Food. Foods.

[6] Picciotti U., Massaro A., Galiano A., Garganese F. (2022). Cheese Fortification: Review and Possible Improvements. Food Rev. Int..

[7] Olson R., Gavin-Smith B., Ferraboschi C., Kraemer K. (2021). Food Fortification: The Advantages, Disadvantages and Lessons from Sight and Life Programs. Nutrients.

[8] Kumar D.S. (2015). Herbal Bioactives and Food Fortification: Extraction and Formulation.

[9] Chadare F.J., Idohou R., Nago E., Affonfere M., Agossadou J., Fassinou T.K., Kénou C., Honfo S., Azokpota P., Linnemann A.R. (2019). Conventional and Food-to-food Fortification: An Appraisal of Past Practices and Lessons Learned. Food Sci. Nutr..

[10] FAO (2022). https://www.fao.org/3/i3396e/i3396e.pdf.

[11] Kamal H., Habib H.M., Ali A., Show P.L., Koyande A.K., Kheadr E., Ibrahim W.H. (2022). Food Waste Valorization Potential: Fiber, Sugar, and Color Profiles of 18 Date Seed Varieties (*Phoenix dactylifera*, L.). J. Saudi Soc. Agric. Sci..

[12] Oladzad S., Fallah N., Mahboubi A., Afsham N., Taherzadeh M.J. (2021). Date Fruit Processing Waste and Approaches to Its Valorization: A Review. Bioresour. Technol..

[13] Taghian Dinani S., van der Goot A.J. (2022). Challenges and Solutions of Extracting Value-Added Ingredients from Fruit and Vegetable by-Products: A Review. Crit. Rev. Food Sci. Nutr..

[14] Mandal D.D., Singh G., Majumdar S., Chanda P. (2022). Challenges in Developing Strategies for the Valorization of Lignin—A Major Pollutant of the Paper Mill Industry. Environ. Sci. Pollut. Res..

[15] Devaux A., Goffart J.-P., Kromann P., Andrade-Piedra J., Polar V., Hareau G. (2021). The Potato of the Future: Opportunities and Challenges in Sustainable Agri-Food Systems. Potato Res..

[16] Silva Pereira Basílio L., Vanz Borges C., Forlan Vargas P. (2020). Potential of Colored Sweet Potato Genotypes as Source of Bioactive Compounds.

[17] Tunio M.H., Gao J., Shaikh S.A., Lakhiar I.A., Qureshi W.A., Solangi K.A., Chandio F.A. (2020). Potato Production in Aeroponics: An Emerging Food Growing System in Sustainable Agriculture Forfood Security. Chil. J. Agric. Res..

[18] Akinlolu-Ojo T., Nwanna E.E., Badejo A.A. (2022). Physicochemical Constituents and Anti-Oxidative Properties of Ripening Hog Plum (*Spondias Mombin*) Fruits and the Quality Attributes of Jam Produced from the Fruits. Meas. Food.

[19] Pérez-Herrera A., Martínez-Gutiérrez G.A., León-Martínez F.M., Sánchez-Medina M.A. (2020). The Effect of the Presence of Seeds on the Nutraceutical, Sensory and Rheological Properties of Physalis Spp. Fruits Jam: A Comparative Analysis. Food Chem..

[20] Alqahtani N.K., Alnemr T.M., Ahmed A.R., Ali S. (2022). Effect of Inclusion of Date Press Cake on Texture, Color, Sensory, Microstructure, and Functional Properties of Date Jam. Processes.

[21] Jati I.R.A.P., Darmoatmodjo L.M.Y.D., Suseno T.I.P., Ristiarini S., Wibowo C. (2022). Effect of Processing on Bioactive Compounds, Antioxidant Activity, Physicochemical, and Sensory Properties of Orange Sweet Potato, Red Rice, and Their Application for Flake Products. Plants.

[22] AOAC Association of Official Analytical Chemists (2012). Official Methods of Analysis.

[23] Habib H.M., Ibrahim W.H. (2011). Nutritional Quality of 18 Date Fruit Varieties. Int. J. Food Sci. Nutr..

[24] Habib H.M., Al Meqbali F.T., Kamal H., Souka U.D., Ibrahim W.H. (2014). Physicochemical and Biochemical Properties of Honeys from Arid Regions. Food Chem..

[25] Habib H.M., El-Fakharany E.M., Kheadr E., Ibrahim W.H. (2022). Grape Seed Proanthocyanidin Extract Inhibits DNA and Protein Damage and Labile Iron, Enzyme, and Cancer Cell Activities. Sci. Rep..

[26] Habib H.M., Theuri S.W., Kheadr E.E., Mohamed F.E. (2017). Functional, Bioactive, Biochemical, and Physicochemical Properties of the Dolichos Lablab Bean. Food Funct..

[27] Habib H.M., El-Fakharany E.M., Souka U.D., Elsebaee F.M., El-Ziney M.G., Ibrahim W.H. (2022). Polyphenol-Rich Date Palm Fruit Seed (*Phoenix dactylifera* L.) Extract Inhibits Labile Iron, Enzyme, and Cancer Cell Activities, and DNA and Protein Damage. Nutrients.

[28] Habib H.M., Theuri S.W., Kheadr E.E., Mohamed F.E. (2017). DNA and BSA Damage Inhibitory Activities, and Anti-Acetylcholinesterase, Anti-Porcine α-Amylase and Antioxidant Properties of Dolichos Lablab Beans. Food Funct..

[29] Pinto D., Moreira M.M., Vieira E.F., Švarc-Gajić J., Vallverdú-Queralt A., Brezo-Borjan T., Delerue-Matos C., Rodrigues F. (2023). Development and Characterization of Functional Cookies Enriched with Chestnut Shells Extract as Source of Bioactive Phenolic Compounds. Foods.

[30] Kavaya R.I., Omwamba M.N., Chikamai B.N., Mahungu S.M. (2019). Sensory Evaluation of Syneresis Reduced Jam and Marmalade Containing Gum Arabic from *Acacia senegal* var. kerensis. Food Nutr. Sci..

[31] Amagloh F.C., Kaaya A.N., Yada B., Chelangat D.M., Katungisa A., Amagloh F.K., Tumuhimbise G.A. (2022). Bioactive Compounds and Antioxidant Activities in Peeled and Unpeeled Sweetpotato Roots of Different Varieties and Clones in Uganda. Future Foods.

[32] Pazos J., Zema P., Corbino G.B., Gabilondo J., Borioni R., Malec L.S. (2022). Growing Location and Root Maturity Impact on the Phenolic Compounds, Antioxidant Activity and Nutritional Profile of Different Sweet Potato Genotypes. Food Chem. Mol. Sci..

[33] Baah R.O., Duodu K.G., Emmambux M.N. (2022). Cooking Quality, Nutritional and Antioxidant Properties of Gluten-Free Maize—Orange-Fleshed Sweet Potato Pasta Produced by Extrusion. LWT.

[34] Gabilondo J., Corbino G., Chludil H., Malec L. (2022). Bioactive Compounds of Two Orange-Fleshed Sweet Potato Cultivars (*Ipomoea Batatas* (L.) Lam.) in Fresh, Stored and Processed Roots. Appl. Food Res..

[35] Rambabu K., Bharath G., Hai A., Banat F., Hasan S.W., Taher H., Mohd Zaid H.F. (2020). Nutritional Quality and Physico-Chemical Characteristics of Selected Date Fruit Varieties of the United Arab Emirates. Processes.

[36] Aleid S.M., Hassan B.H., Almaiman S.A., Al-Kahtani S.H., Ismail S.M. (2014). Microbial Loads and Physicochemical Characteristics of Fruits from Four Saudi Date Palm Tree Cultivars: Conformity with Applicable Date Standards. Food Nutr. Sci..

[37] Derouich M., Meziani R., Bourkhis B., Filali-Zegzouti Y., Alem C. (2020). Nutritional, Mineral and Organic Acid Composition of Syrups Produced from Six Moroccan Date Fruit (*Phoenix dactylifera* L.) Varieties. J. Food Compos. Anal..

[38] Alem C., Ennassir J., Benlyas M., Mbark A.N., Zegzouti Y.F. (2017). Phytochemical Compositions and Antioxidant Capacity of Three Date (*Phoenix dactylifera* L.) Seeds Varieties Grown in the South East Morocco. J. Saudi Soc. Agric. Sci..

[39] Hmidani A., Bourkhis B., Khouya T., Ramchoun M., Filali-Zegzouti Y., Alem C. (2020). Phenolic Profile and Anti-Inflammatory Activity of Four Moroccan Date (*Phoenix dactylifera* L.) Seed Varieties. Heliyon.

[40] Sanoussi A.F., Adjatin A., Dansi A., Adebowale A., Sanni L.O., Sanni A. (2016). Mineral Composition of Ten Elites Sweet Potato (*Ipomoea Batatas* [L] Lam) Landraces of Benin. Int. J. Curr. Microbiol. Appl. Sci..

[41] Franková H., Musilová J., Árvay J., Šnirc M., Jančo I., Lidiková J., Vollmannová A. (2022). Changes in Antioxidant Properties and Phenolics in Sweet Potatoes (*Ipomoea batatas* L.) Due to Heat Treatments. Molecules.

[42] Al-Mssallem M.Q., Alqurashi R.M., Al-Khayri J.M. (2019). Bioactive Compounds of Date Palm (*Phoenix dactylifera* L.). Bioactive Compounds in Underutilized Fruits and Nuts.

[43] Al-Shwyeh H. (2019). Date Palm (*Phoenix dactylifera* L.) Fruit as Potential Antioxidant and Antimicrobial Agents. J. Pharm. Bioallied Sci..

[44] Chaari A., Abdellatif B., Nabi F., Khan R.H. (2020). Date Palm (*Phoenix dactylifera* L.) Fruit’s Polyphenols as Potential Inhibitors for Human Amylin Fibril Formation and Toxicity in Type 2 Diabetes. Int. J. Biol. Macromol..

[45] Kim M.Y., Lee B.W., Lee H., Lee Y.Y., Kim M.H., Lee J.Y., Lee B.K., Woo K.S., Kim H. (2019). Phenolic Compounds and Antioxidant Activity in Sweet Potato after Heat Treatment. J. Sci. Food Agric..

[46] Kwatra B. (2020). A review on potential properties and therapeutic applications of grape seed extract. Artic. World J. Pharm. Res..

[47] Manca M.L., Casula E., Marongiu F., Bacchetta G., Sarais G., Zaru M., Escribano-Ferrer E., Peris J.E., Usach I., Fais S. (2020). From Waste to Health: Sustainable Exploitation of Grape Pomace Seed Extract to Manufacture Antioxidant, Regenerative and Prebiotic Nanovesicles within Circular Economy. Sci. Rep..

[48] Gülcü M., Uslu N., Özcan M.M., Gökmen F., Özcan M.M., Banjanin T., Gezgin S., Dursun N., Geçgel Ü., Ceylan D.A. (2019). The Investigation of Bioactive Compounds of Wine, Grape Juice and Boiled Grape Juice Wastes. J. Food Process Preserv..

[49] Sridhar K., Charles A.L. (2019). In Vitro Antioxidant Activity of Kyoho Grape Extracts in DPPH and ABTS Assays: Estimation Methods for EC50 Using Advanced Statistical Programs. Food Chem..

[50] Rousserie P., Lacampagne S., Vanbrabant S., Rabot A., Geny-Denis L. (2020). Influence of Berry Ripeness on Seed Tannins Extraction in Wine. Food Chem..

[51] Mohd Naeem M.N., Mohd Fairulnizal M.N., Norhayati M.K., Zaiton A., Norliza A.H., Wan Syuriahti W.Z., Mohd Azerulazree J., Aswir A.R., Rusidah S. (2017). The Nutritional Composition of Fruit Jams in the Malaysian Market. J. Saudi Soc. Agric. Sci..

[52] Chalchisa T., Zegeye A., Dereje B., Tolesa Y. (2022). Effect of Sugar, Pectin, and Processing Temperature on the Qualities of Pineapple Jam. Int. J. Fruit Sci..

[53] Bikila A.M., Tola Y.B., Esho T.B., Forsido S.F. (2022). Anchote (*Coccinia abyssinica* [Lam.] Cogn.) Powder, an Underutilized Indigenous Crop, as a Substitute to Commercial Pectin in the Production of Strawberry Jam. Heliyon.

[54] Teixeira F., dos Santos B.A., Nunes G., Soares J.M., do Amaral L.A., de Souza G.H.O., de Resende J.T.V., Menegassi B., Rafacho B.P.M., Schwarz K. (2020). Addition of Orange Peel in Orange Jam: Evaluation of Sensory, Physicochemical, and Nutritional Characteristics. Molecules.

[55] Aghajanzadeh S., Ziaiifar A.M., Verkerk R. (2021). Effect of Thermal and Non-Thermal Treatments on the Color of Citrus Juice: A Review. Food Rev. Int..

